# The geriatric nutrition risk index versus the mini-nutritional assessment short form in predicting postoperative delirium and hospital length of stay among older non-cardiac surgical patients: a prospective cohort study

**DOI:** 10.1186/s12877-020-1501-8

**Published:** 2020-03-17

**Authors:** Yanli Zhao, Ning Ge, Dongmei Xie, Langli Gao, Yanyan Wang, Yulin Liao, Jirong Yue

**Affiliations:** 1 Department of Geriatrics and National Clinical Research Center for Geriatrics, West China Hospital of Sichuan University, Chengdu, 610041 Sichuan China; 2 Department of Respiratory Diseases, First Affiliated Hospital of Guangxi Medical University, Nanning, 530021 Guangxi China

**Keywords:** Geriatric nutritional risk index, Mini-nutritional assessment short form, Older people, Postoperative delirium, Length of stay, Non-cardiac surgery

## Abstract

**Backgrounds:**

Malnutrition has been shown to be associated with poor prognosis in older surgical patients. Several tools are available for detecting malnutrition. But little is known about their ability to assess risks of postoperative adverse outcomes. The study aimed to compare the ability of the Geriatric Nutritional Risk Index (GNRI) and the Mini-Nutritional Assessment Short Form (MNA-SF) in predicting postoperative delirium (POD) and length of stay (LOS) among older non-cardiac surgical patients.

**Methods:**

Prospective study of 288 older non-cardiac surgical patients from *the West China Hospital of Sichuan University*. Preoperative nutritional status was assessed using the GNRI and MNA-SF, and patients were followed for the occurrence of POD and LOS. Multivariable logistic regression and linear regression analyses were used to identify predictors of these outcomes. The relative performance of the GNRI and MNA-SF as predictors of these outcomes were determined by Receiver Operating Characteristic curves (ROC) analyses and the area under the curve (AUC).

**Results:**

Multivariable analysis revealed that preoperative malnutrition by the MNA-SF was significantly associated with POD. Linear regression analysis showed that preoperative low/high nutritional risk of the GNRI and malnutrition by the MNA-SF were independent predictors of prolonged LOS. Moreover, the area under the curve (AUC) of MNA-SF scores for POD was better than GNRI scores (AUC = 0.718, 95%CI: 0.64–0.80, *P* < 0.001 vs AUC = 0.606, 95%CI: 0.52–0.69, *P =* 0.019; Delong’s test, *P* = 0.006), but the AUC of GNRI scores and MNA-SF scores have no significant difference when predicting prolonged LOS (AUC = 0.611, 95%CI: 0.54–0.69, *P =* 0.006 vs AUC = 0.533, 95%CI: 0.45–0.62, *P =* 0.421; Delong’s test, *P* = 0.079).

**Conclusion:**

The MNA-SF was more effective than the GNRI at predicting the development of POD, but the two nutrition screening methods have similar performance in predicting prolonged LOS among older non-cardiac surgical patients.

## Background

The rapid aging of the general population is resulting in a greater number of older patients in need of surgery. Malnutrition is a common comorbidity in surgical patients. Advanced age, chronic diseases, reliance upon a large number of drugs, low nutrient intake, reduced appetite, and psychological conditions are risk factors for the development of nutritional deficiencies [1, 2]. The prevalence of malnutrition in geriatric hospitalized patients has been estimated to range from 30 to 60% depending on the population studied and the applied assessment tools [3, 4]. Despite these high rates of malnutrition, this issue has not received sufficient clinical attention [5]. The presence of malnutrition is associated with adverse clinical outcomes, including a higher rate of delirium, prolonged length of stay, morbidity, mortality and increase of healthcare costs [6–8]. Furthermore, several studies have found that nutritional intervention can mitigate the risk of delirium and prolonged hospitalization [9, 10]. Therefore, early nutritional screening in hospitalized patients is important for estimating the risk of nutrition-related complications, especially delirium and length of hospital stay.

There are currently multiple different screening tools available for assessing nutritional status in the older people. Of these tools, the Mini Nutritional Assessment-Short Form (MNA-SF) is recommended by the European Society for Clinical Nutrition and Metabolism (ESPSN), as it has been validated for the diagnosis of malnutrition and for prediction of clinical outcomes [11, 12]. Recently, a novel screening tool, the Geriatric Nutritional Risk Index (GNRI), has been proposed [13]. As this screening method is dependent upon objective measurements that do not require patient cooperation, it can be applied in all clinical settings [14, 15]. The validity of the GNRI for the prediction of short and long-term outcomes has been clearly demonstrated in previous studies [16, 17]. To date, some studies have compared the ability of different nutritional screening tools to assess malnutrition status, hospital length of stay, mortality, and infection-associated complications in hospitalized patients [17–19]. However, studies comparing the ability of the GNRI and MNA-SF in predicting postoperative delirium (POD) and length of hospital stay (LOS) are still scarce.

Thus, the aim of our study was to compare the GNRI with the MNA-SF regarding its ability to predict POD and LOS in older non-cardiac surgical patients.

## Methods

### Study design and population

*This prospective cohort study was conducted in the West China Hospital of Sichuan University from April to June of 2015. Eligible patients were 70 years or older,* scheduled for elective *non-cardiac surgery, and* had an anticipated length of stay of at least 3 days*. Exclusion criteria included:* (1) *severe hearing impairment,* (2) inability to communicate because of severe dementia or psychiatric illness*,* (3) a terminal condition with life expectancy of less than 6 months (eg, metastatic cancer, pancreatic cancer, or receiving end-of-life care)*,* (4) *the presence of delirium* at baseline*.*

The study was approved by the Institutional Review Boards of West China Hospital, Sichuan University, and was carried out according to the principles of the Declaration of Helsinki. All the participants provided written informed consent.

### Data collection and sample size

All patients were preoperatively assessed by trained research nurses within 48 h of admission, and the following data were collected: age, gender, and type of surgery (orthopedic, general, thoracic). Delirium was initially screened using the Confusion Assessment Method (CAM) on admission. Comorbidities were evaluated on admission using the CCI (Charlson Comorbidity Index) [20]. Functional status was evaluated using the Barthel Index [21]*.* Preoperative pain was measured using the Facial Scale (range 0–10) via patient interview [22]. T*he presence of depressive symptoms was assessed using the 15-item version of the validated Geriatric Depression Scale (GDS-15)* [23]*.*

Previous results suggested that the incidence of POD in older patients was 13–50% [24]. We hypothesized that the incidence rate in our study was 20% and the AUC was not less than 0.7. Assuming a type I error of 5%, a type II error of 10%, and taking design into account, we estimated the sample size to be 135. Allowing for 20% attrition, we increased the sample size to 168.

### Nutritional assessment

*The GNRI and MNA-SF were used to assess preoperative nutritional status. The GNRI, which was adapted from the Nutritional Risk Index (NRI) designed by Buzby* et al. [25]*, is a simple nutritionl screening tool to evaluate nutritional-related complications. The index was calculated as follows* [13]*: GNRI =* 1.489 × serum albumin (g/L) + 41.7 × present weight/ideal weight (kg). Ideal body weight was derived according to the following equations of Lorentz [13]: ideal weight for men = 0.75 × height (cm) – 62.5, ideal weight for women = 0.60 × height (cm) – 40*.* Unlike the original categorization in four classes proposed by Bouillanne et al. [13]. *The participants in our study were stratified into three categories similar to previous study: no risk (GNRI >* 98*), low risk (92–98), severe/moderate risk (*GNRI < 92*)* [17]*. We merged the category of* severe risk (GNRI< 82) and moderate risk (GNRI 82 to < 92) into one single category, as these two categories were associated with a similar increased odds of complications [17]. *The MNA-SF is a validated, sensitive, reliable screening tool which consists of six domains: appetite or eating problems in the past 3 months, weight loss in the past 3 months, mobility impairment, acute illness/stress, dementia or depression, and BMI. Total scores of MNA-SF range from 0 to 14, and patients were divided into the following three categories according to the following cut-offs: well-nourished* (12–14)*, risk of malnutrition* (8–11)*, malnourished (0–7)* [26]*.*

### Outcomes

*All patients were followed for the occurrence of POD and LOS.* The CAM was used daily by the trained research assessors for delirium within 7 days after surgery. The CAM is based on the following four features [27]: (i) acute onset and fluctuating course; (ii) inattention; (iii) disorganized thinking; (iv) altered level of consciousness. A positive diagnosis of delirium required the presence of both items (i) and (ii), and either item (iii) or (iv). LOS was defined as the number of days in the hospital from the day of admission to the day of discharge. Prolonged LOS was defined as LOS beyond the 75th percentile of its distribution (computed to ≥22 days in our study) [28].

### Statistical analysis

Continuous variables were expressed as means with the standard deviation (SD) for normally distributed data, and as medians with the interquartile range (IQR) for non-normally distributed data. ANOVAs and Kruskall-Wallis tests were used for between-group comparisons of continuous variables with normal and non-normal distributions, respectively. Categorical variables were expressed as the number of cases and percentages, and were compared using *Chi-squared tests*.

A multivariable logistic regression model was performed to investigate the association between the two screening methods and POD, and a linear regression model was used for LOS. The *logistic regression model was adjusted for age, sex,* preoperative pain, *depression, Barthel Index and CCI*. The *linear regression model was adjusted for age, sex, Barthel Index* and preoperative pain. The GNRI and MNA-SF were included in regression models as continuous and *categorical variables respectively. To fulfill the purpose of the study*, *the discriminative ability of each nutritional screening tool for the outcomes* was assessed by the Receiver Operating Characteristic (ROC) curves and the area under the curve (AUC). Comparisons between AUCs were performed by Delong’s test [29]*.* Furthermore, *the sensitivity, specificity and* likelihood ratios *(positive and negative) of different GNRI and MNA-SF cut-offs were calculated.*

*Statistical analyses were performed using IBM SPSS version 21.0 and MedCalc version 19.1.* A *P* value < 0.05 was considered statistically significant.

## Results

### Baseline characteristic of patients

A total of 348 patients were admitted to our hospital. 28 and 32 patients were excluded due to cancelling scheduled surgery and incomplete data, respectively. Finally, 288 subjects were included in the analyses. The median age of these patients was 74 years (IQR 72–28), and 148 patients (51.4%) were male. Of the overall population, 49 (17%) developed POD, and median LOS was 14 days (IQR 10–21). In our study, the number of patients who underwent general, orthopedic and thoracic surgery were 189 (65.6%), 71 (24.7%) and 28 (9.7%), respectively.

### The characteristics of the population as determined by the MNA-SF and GNRI

The characteristics of the patients screened by the MNA-SF and GNRI are shown in Tables 1 and 2. According to the GNRI, 29.5 and 15.6% of patients were low risk and high risk, respectively. Based on the MNA-SF, 34 and 14.2% of patients were at risk of malnutrition and malnourished, respectively. There were significant differences in the Barthel Index, POD incidence, and LOS among different GNRI and MNA-SF categories. By a post-hoc comparison, we found that albumin levels was significantly lower in subjects who were malnourished and at risk of malnutrition compared to those who were well-nourished according to the MNA-SF.

### Multivariable logistic regression and linear regression analysis

In the multivariable model, the malnourished category of the MNA-SF was independent risk factor for POD after adjustment of age, sex, preoperative pain, *depression, Barthel Index and CCI*, while the GNRI was not predictor for POD (Table 3). In the linear regression, prolonged LOS was significantly associated with low and high risk of the GNRI, but only with malnourished category by the MNA-SF after adjustment of age, sex, Barthel Index and preoperative pain (Table 3). When modeled as a continuous variable, MNA-SF was independent predictor of prolonged LOS and POD, while the GNRI was only significantly correlated with LOS.

### ROC curve analysis

**Fig. 1:**
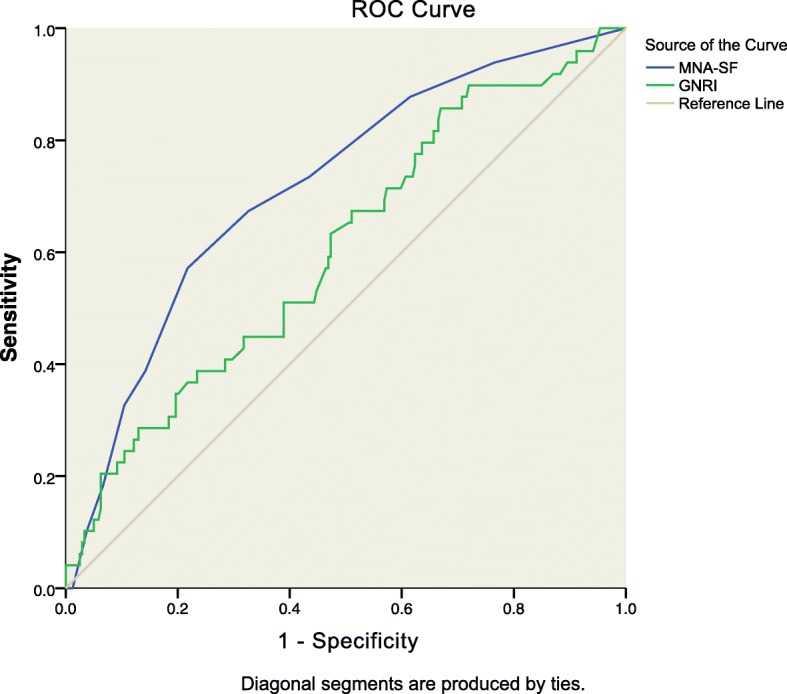
Receiver operator curve (ROC) of the GNRI and MNA-SF scores for postoperative delirium

**Fig. 2:**
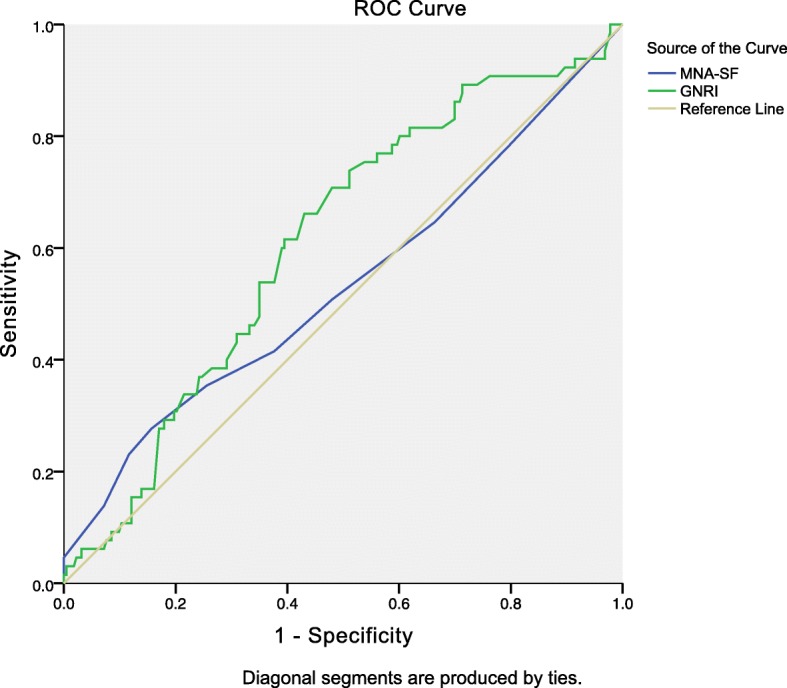
Receiver operator curve (ROC) of the GNRI and MNA-SF scores for prolonged length of stay

Based on the ROC curve analyses and Delong’s test, MNA-SF scores showed higher AUC in predicting POD than GNRI scores (Table 4, Fig. 1). In addition, the AUC of MNA-SF scores was significantly higher than GNRI scores (Delong’s test, *P* = 0.006). Although the AUC of GNRI scores for prolonged LOS was better than MNA-SF scores (Table 4, Fig. 2), there was no significant difference in determining prolonged LOS (Delong’s test, *P* = 0.079). As shown in Table 4, both MNA-SF < 8 and GNRI< 92 exhibited satisfactory specificity values (>60%) in predicting POD and prolonged LOS. However, the sensitivity values of the two categories were below adequate (< 80%).

**Table 1:** Characteristics of the studied population according to the Geriatric Nutritional Risk Index (GNRI)

Characteristic	Total	High Risk < 92 (*n* = 45)	Low Risk 92–98 (*n* = 85)	No Risk > 98 (*n* = 158)	*P*-value ^a^
Age (years), median (IQR)	74 (72–78)	75 (73–79)	75 (72–78)	74 (71–77)	0.086
Male gender, *n* (%)	148 (51.4)	24 (53.3)	36 (42.4)	88(55.7)	0.134
Preoperative pain, *n* (%)	169 (58.7)	26 (57.8)	56 (65.9)	87 (55.1)	0.261
Depression, *n* (%)	19 (6.6)	4 (8.9)	5 (5.9)	10 (6.3)	0.79
CCI, *n* (%)					
Mild (≤2)	214 (74.3)	34 (75.6)	58 (68.2)	122 (77.2)	
Moderate (3, 4)	52 (18.1)	7 (15.6)	21 (24.7)	24 (15.2)	0.546
Severe (≥5)	22 (7.6)	4 (8.9)	6 (7.1)	12 (7.6)	
GNRI score, mean ± SD	98.98 ± 8.46	85.09 ± 5.45 ^b^	95.12 ± 2.17 ^b^	105.01 ± 4.49 ^b^	<0.001
MNA-SF score, med (IQR)	12 (9–13)	9 (7–11)	10 (8–12)	12 (11–14)	<0.001
Barthel Index, median (IQR)	100 (90–100)	95 (72–100)	100 (87–100)	100 (95–100)	0.010
Postoperative delirium, *n* (%)	49 (17.0)	14 (31.1)	11 (12.9)	24 (15.2)	0.021
Length of stay, med (IQR)	14 (21–10)	17 (12–21)	17 (12–23)	13 (9–18)	<0.001

*BMI* body mass index, *CCI* Charlson Comorbidity Index, *MNA-SF* Mini Nutritional Assessment-Short Form.

Notes: ^a^*p* values according to ANOVA, Kruskall-Wallis or Chi-square tests;

^b^ Significantly different from the other groups by post-hoc comparison

**Table 2:** Characteristics of the studied population according to the Mini-Nutritional Assessment Short Form (MNA-SF)

Characteristic	Malnourished ≤ 7 (*n* = 41)	At risk 8–11 (*n* = 98)	Well nourished 12–14 (*n* = 149)	*P*-value^a^
Age (years), median (IQR)	76 (73–78)	75.5 (72–78)	73 (71–77)	0.078
Male gender, *n* (%)	22 (53.7)	49 (50.0)	77 (51.7)	0.921
Preoperative pain, *n* (%)	30 (24.1)	59 (60.2)	80 (53.7)	0.075
BMI (kg/m^2^), mean ± SD	19.45 ± 2.55 ^b^	21.42 ± 3.07 ^b^	23.70 ± 2.88 ^b^	<0.001
Albumin (g/L), mean ± SD	36.93 ± 5.13 ^b^	39.79 ± 4.73 ^b^	41.21 ± 4.48 ^b^	<0.001
MNA-SF score, med (IQR)	6 (5–7) ^b^	10 (9–11) ^b^	13 (12–14) ^b^	<0.001
Barthel Index, median (IQR)	95 (77–100)	100 (88–100)	100 (95–100)	0.010
Postoperative delirium, *n* (%)	16 (39.0)	20 (20.4)	13 (8.7)	0.004
Length of stay, med (IQR)	19 (14–23)	14 (10–19)	14 (10–20)	0.022

*BMI* body mass index, *CCI* Charlson Comorbidity Index, *GNRI* Geriatric Nutritional Risk Index.

Notes: ^a^*p* values according to ANOVA, Kruskall-Wallis or Chi-square tests;

^b^ Significantly different from the other groups by post-hoc comparison

**Table 3:** Multivariable logistic regression and linear regression analyses for the occurrence of postoperative delirium and length of stay

	Postoperative delirium		Length of stay	
	Adjusted OR (95% CI)^a^	*P*-value	β (95% CI)^b^	*P*-value
**GNRI categories**^c^				
No risk (>98)	Reference		Reference	
Low risk (92–98)	0.61 (0.26–1.43)	0.255	4.91 (2.43–7.39)	< 0.001
High risk (<92)	2.22 (0.92–5.37)	0.077	4.10 (0.95–7.25)	0.011
**GNRI scores**^d^	0.96 (0.92–1.00)	0.077	−0.24 (−0.37 to − 0.11)	< 0.001
**MNA-SF categories**^c^				
Well nourished (12–14)	Reference		Reference	
At risk (8–11)	1.95 (0.86–4.39)	0.109	0.87 (−1.55 to 3.29)	0.48
Malnourished (≤7)	4.06 (1.62–10.18)	0.003	4.23 (0.91–7.54)	0.013
**MNA-SF scores**^d^	0.82 (0.72–0.92)	0.001	−0.52 (− 0.93 to − 0.11)	0.014

*OR* odds ratio, *CI* confidence interval, *GNRI* Geriatric Nutritional Risk Index, *MNA-SF* Mini-Nutritional Assessment Short Form, *CCI* Charlson Comorbidity Index.

Notes: ^a^ Adjusted for age, sex, CCI, depression, Barthel Index and preoperative pain in multivariable logistic regression model;

^b^ Adjusted for age, sex, Barthel Index and preoperative pain in linear regression model;

^c^ Modeled as categories variables;

^d^ Modeled as continuous variables

**Table 4:** Sensitivity, specificity, positive likelihood ratios (PLR), negative likelihood ratios (NLR) and area under the curve (AUC) of nutritional screening tools

	Sensitivity	Specificity	PLR	NLR	AUC (95% CI)	*P* (AUC)
**Postoperative delirium**						
GNRI						
Scores <92	28.6%	87.0%	2.20	0.82	0.606 (0.52–0.69)	0.019
Scores ≤98	51.0%	56.1%	1.16	0.87		
MNA-SF						
Scores <8	32.7%	89.5%	3.11	0.75	0.718 (0.64–0.80)	<0.001
Scores <12	73.5%	56.9%	1.71	0.47		
**Prolonged length of stay**						
GNRI						
Scores <92	16.9%	84.8%	1.11	0.98	0.611 (0.54–0.69)	0.006
Scores ≤98	61.5%	59.6%	1.52	0.65		
MNA-SF						
Scores <8	23.1%	88.3%	1.97	0.87	0.533 (0.45–0.62)	0.421
Scores <12	50.8%	52.5%	1.07	0.94		

*CI* confidence interval, *GNRI* Geriatric Nutritional Risk Index, *MNA-SF* Mini-Nutritional Assessment Short Form.

## Discussion

*Early and accurate identification of patients at risk of malnutrition-associated complications could effectively guide surgical practices. Although there are number of malnutrition assessment tools available designed to determine the risk of postoperative adverse outcomes, there are at present few studies* evaluating the validity of the MNA-SF and the GNRI as predictors of nutrition-related morbidity in a surgical setting*.* To the best of our knowledge, this is the first prospective *study to compare the ability of the GNRI and MNA-SF in predicting POD and LOS among* older patients undergoing non-cardiac surgery. Our results revealed that the MNA-SF seemed to be better at predicting POD, whereas the MNA-SF and the GNRI have similar ability in predicting the risk of POD among older non-cardiac surgical patients.

In this study, the prevalence of high risk of malnutrition determined by the GNRI was lower than that reported by Duran et al. [30], in which 32.5% of 40 elderly patients in acute geriatric ward were at high nutritional risk. *Patients in the* acute geriatric ward were more likely to be have severe and acute illness than surgical patients, which may explain these differences. The MNA-SF detected malnutrition in 14.2% of patients in the current study. Another study found a similar prevalence of malnutrition by the MNA-SF [31].

Serum albumin is generally considered as a crude indicator for nutritional status, particularly among patients with chronic conditions; a low level of serum albumin could indicate either poor nutritional status or inflammatory status or both [32]. Previous studies have demonstrated that the MNA-SF and the GNRI were more suitable for assessing nutritional status than serum albumin [13, 33]. In addition, the GNRI and MNA-SF had better performance in predicting adverse outcomes than serum albumin [34, 35]. Thus, more comprehensive and systematic nutritional screening methods, such as the GNRI and the MNA-SF, are needed to evaluate nutritional status or nutrition-related complications among surgical patients.

According to the present results, the MNA-SF outperformed GNRI in predicting POD among elderly non-cardiac surgical patients. To date, only one study by Sugita, et al. compared different screening tools, including the GNRI, PNI and CONUT, for prediction of delirium in coronary intensive care unit patients and they observed no a significant association between GNRI and delirium [36], which is similar to our study. Recently, Chu et al. and Mazzola et al. used the MNA-SF as a screening tool among orthopedic surgical patients and detected a significant association between MNA-SF and POD [31, 37]. In addition, previous studies have found that the nutritional intervention could reduce the incidence of postoperative delirium and shorten its duration in older surgical patients [10, 38, 39]. Our results were consistent with these, and confirmed the value of the MNA-SF as a predictor of POD.

The superiority of the MNA-SF as a predictor of POD may be explained by the fact that it incorporates neuropsychological, functional and psychological parameters, all of which are risk factors for the development of delirium. Although depression and dementia, included in the items of the MNA-SF, are also part of the CCI and GDS-15. We have excluded patients with the severe dementia on admission and the number of patients with severe depression is low in our study. Moreover, unlike the dementia and mild/moderate depression items of the MNA-SF, severe dementia and depression should be diagnosed by the specialist.

The GNRI and the MNA-SF have similar performance in predicting prolonged LOS in the present study. This is different from findings reported in Abd-EL-Gawad et al., in which the GNRI was found more effective than the MNA in the evaluation of prolonged hospitalization [19]. Recently, many studies have used nutritional screening tools to predict hospitalization period [40–42]. There is accumulating evidence that early nutritional screening in older patients who might benefit from nutritional treatment may result in a shorter LOS [6, 8, 9]. These studies support that malnutrition is useful for predicting LOS. However, the causal relation between nutritional status and LOS remains unclear; rather, length of hospital stay may reflect the severity of underlying disease.

Indeed, the MNA-SF and GNRI are relatively simple screening tools that can be rapidly applied to clinical practice. Advantages of the MNA-SF are its high sensitivity in regard to nutritional assessment and the lack of requirement for biochemical tests. However, it cannot be used in patients receiving parenteral nutrition or who have poor cognitive function [3]. The GNRI was designed to overcome the subjective bias of the MNA and the difficulties in acquiring usual weight and standing height [13, 15]. It may be useful in surgical patients with cognitive impairment because it is an objective index based only on weight, height, and serum albumin levels [13]. Further studies comparing the GNRI and the MNA-SF as predictors of adverse outcomes are required to validate their utility.

This study has several limitations. First, this is a single-center study with a small sample size, the results may not represent a general population of older surgical patients. Multicenter and larger studies are required in the future. Second, the 7-day duration of screening for delirium was chosen to balance the peak days of delirium onset in the population (POD occurred 1–3 days after surgery) against the practical constraints of our resources. Third, due to realistic constraints of our resources, intraoperative data and postoperative data in Intensive Care Units were not included in our study. Fourth, Patients with risk of malnutrition by the MNA-SF were not screened using the MNA owing to practical constraints. The prevalence of malnutrition may be underestimated. Fifth, we did not collect information regarding the occurrence of in-hospital mortality and assessment of functional status at discharge.

## Conclusion

The current study demonstrated that the MAN-SF is more reliable as a means of evaluating patients for the development of POD, whereas the MNA-SF and the GNRI have similar performance in predicting prolonged LOS. Present results may help clinicians to choose appropriate nutrition screening method to predict different outcomes. It also highlights the importance of early detection and timely intervention for patients who are at risk of undernutrition, in order to prevent negative postoperative outcomes. In the future, more studies comparing the ability of different nutritional screening tools in predicting adverse outcomes among surgical patients are needed.

## Data Availability

The datasets used for the current study are available from the corresponding author upon reasonable request.

## Abbreviations

CAM: Confusion Assessment Method
CCI: Charlson Comorbidity Index
GNRI: Geriatric Nutritional Risk Index
LOS: length of stay
MNA-SF: Mini Nutritional Assessment Short Form
POD: postoperative delirium
